# How to Evaluate Cardiac Autonomic Modulation

**DOI:** 10.5935/abc.20180127

**Published:** 2018-07

**Authors:** Esteban W. Rivarola, Mauricio I. Scanavacca

**Affiliations:** Instituto do Coração - Universidade de São Paulo, São Paulo, SP - Brazil

**Keywords:** Autonomic Nervous System Diseases, Heart Rate, Diabetes Mellitus, Nonlinear Dynamics, Primary Dysautonomias, Diabetic Neuropathies/prevention

Cardiac autonomic nervous system dysfunction has been implicated in several different pathological scenarios with a wide range of clinical relevance and risk. Early detection of autonomic changes, either provoked for therapeutic purposes^1^ or as a complication of a primary disorder, such as diabetes mellitus (DM) is of essence for the best management of patients.

Classic (linear) analysis of heart rate variability (HRV) has been routinely used to assess autonomic behavior in diabetic patients in order to promptly detect neuropathy,^2^ one of the most common and overlooked complications and a significant cardiovascular risk factor.

As the linear analysis provides important data, non-linear indexes of HRV have been proposed as well, emerging as potential ancillary tools to investigate dysautonomia in type 1 and 2 DM. In the paper “Nonlinear Dynamics in young people with diabetes”,^3^ the authors compared linear and nonlinear indexes and studied their correlation. While symbolic analysis presented partial correlation with linear methods, Shannon entropy index was similar in DM individuals and controls, and these findings raise two important issues:

1. What could be the clinical value of determining the complexity and randomness of HRV by nonlinear methods?
2. Are they sensitive and efficient?

Several authors agreed that linear indexes (time and frequency domains) are simple and reproducible methods to evaluate the cardiac autonomic system and are consistently reduced in diabetic patients.^2-7^ The observed lack of correlation of nonlinear methods with standard measures may imply low sensitivity, and it stands in disagreement with Javorka et al.,^4^ which claim that “The complexity of HRV appears to be even more affected (in DM patients) than the magnitude of HRV that is commonly assessed by cardiac autonomic tests.”

On the other hand, a perfect correlation between nonlinear techniques and standard HRV measures would provide only limited additional diagnostic information. In fact, previous authors verified that linear HRV indexes performed even better than most complexity measures in discriminating DM patients from controls.^4^

So, where do we stand on the noninvasive diagnosis of dysautonomia?

To the best of our knowledge, time and frequency domain indexes remain the most accepted and used methods to assess HRV. Nonlinear measures are potential tools, but to reach optimal HRV assessment, the methods must be standardized: it is possible to find studies with 24-hour,^8^ medium-term (~1h),^4,7^ ultra short-term (<5 min)^9^ and short-term (from 5-10 min)^2,5,6,10^ data recording indexes, all of them dealing with noninterchangeable information.

Nonlinear methods’ contribution to evaluate diabetic autonomic system dysfunction is yet to be demonstrated by large-scale comparison studies. Once complexity evaluation proves its value, however, one last question will remain: to what extent would it help patients prevent diabetic neuropathy progression?
